# Development of brain-penetrable antibody radioligands for *in vivo* PET imaging of amyloid-β and tau

**DOI:** 10.3389/fnume.2023.1173693

**Published:** 2023-05-04

**Authors:** Vinay Banka, Andrew Kelleher, Dag Sehlin, Greta Hultqvist, Einar M. Sigurdsson, Stina Syvänen, Yu-Shin Ding

**Affiliations:** ^1^ Department of Radiology, New York University School of Medicine, New York, NY, United States; ^2^ Department of Public Health and Caring Sciences, Uppsala University, Uppsala, Sweden; ^3^ Department of Pharmacy, Uppsala University, Uppsala, Sweden; ^4^ Department of Psychiatry, New York University School of Medicine, New York, NY, United States; ^5^ Department of Neuroscience and Physiology, New York University School of Medicine, New York, NY, United States

**Keywords:** antibody, transferrin receptor, blood–brain barrier, PET, [18F]SFB, tau, Aβ, AD

## Abstract

**Introduction:**

Alzheimer's disease (AD) is characterized by the misfolding and aggregation of two major proteins: amyloid-beta (Aβ) and tau. Antibody-based PET radioligands are desirable due to their high specificity and affinity; however, antibody uptake in the brain is limited by the blood–brain barrier (BBB). Previously, we demonstrated that antibody transport across the BBB can be facilitated through interaction with the transferrin receptor (TfR), and the bispecific antibody-based PET ligands were capable of detecting Aβ aggregates via *ex vivo* imaging. Since tau accumulation in the brain is more closely correlated with neuronal death and cognition, we report here our strategies to prepare four F-18-labeled, specifically engineered bispecific antibody probes for the selective detection of tau and Aβ aggregates to evaluate their feasibility and specificity, particularly for *in vivo* PET imaging.

**Methods:**

We first created and evaluated (via both *in vitro* and *ex vivo* studies) four specifically engineered bispecific antibodies, by fusion of single-chain variable fragments (scFv) of a TfR antibody with either a full-size IgG antibody of Aβ or tau or with their respective scFv. Using [^18^F]SFB as the prosthetic group, all four ^18^F-labeled bispecific antibody probes were then prepared by conjugation of antibody and [^18^F]SFB in acetonitrile/0.1 M borate buffer solution (final pH ∼ 8.5) with an incubation of 20 min at room temperature, followed by purification on a PD MiniTrap G-25 size-exclusion gravity column.

**Results:**

Based on both *in vitro* and *ex vivo* evaluation, the bispecific antibodies displayed much higher brain concentrations than the unmodified antibody, supporting our subsequent F18-radiolabeling. [^18^F]SFB was produced in high yields in 60 min (decay-corrected radiochemical yield (RCY) 46.7 ± 5.4) with radiochemical purities of >95%, confirmed by analytical high-performance liquid chromatography (HPLC) and radio-TLC. Conjugation of [^18^F]SFB and bispecific antibodies showed a conversion efficiency of 65%-83% with radiochemical purities of 95%–99% by radio-TLC.

**Conclusions:**

We successfully labeled four novel and specifically engineered bispecific antibodies with [^18^F]SFB under mild conditions with a high RCY and purities. This study provides strategies to create brain-penetrable F-18 radiolabeled antibody probes for the selective detection of tau and Aβ aggregates in the brain of transgenic AD mice via *in vivo* PET imaging.

## 1. Introduction

Amyloid-β (Aβ) and tau protein are the principal elements of plaques and tangles, respectively. Since the discovery of Aβ and tau, the development of diagnostic and therapeutic strategies for Alzheimer’s disease (AD) has initially focused on Aβ (1–3), but tau has received more attention in recent years, in part because of the failure of several Aβ-targeting treatments in clinical trials (1, 4–6) and a stronger association between tau pathology and cognitive decline (1, 3).

Antibody-based PET radioligands are more desirable due to their specificity and high affinity; however, in the central nervous system (CNS), antibody uptake is limited due to their inability to cross the blood–brain barrier (BBB). Various strategies need to be implemented to enhance the brain uptake of a radiolabeled antibody in order to achieve PET imaging.

Previously, we successfully conjugated mAb158 (an Aβ antibody) to a transferrin receptor (TfR) antibody to enable receptor-mediated transcytosis across the BBB (7). Our *ex vivo* imaging study using radiolabeled bispecific antibody ligands in two different mouse models with Aβ pathology visualized Aβ in the brain. The PET signal increases with age and closely correlates with brain Aβ levels, demonstrating that bispecific antibody PET ligands can be successfully used for the brain imaging of Aβ pathology (8–10). We have also previously reported on the diagnostic imaging potential of the tau ligands 6B2G12 and scFv235 in tauopathy mice, e.g., in vivo imaging system (IVIS) imaging, in which they selectively detect pathological tau lesions *in vivo* (11). The fact that tau accumulation in the brain is more closely correlated with neuronal death and ultimately loss of cognitive function (12–14) makes it critical to develop ^18^F-radiolabeled bispecific tau antibody ligands for *in vivo* imaging of tau for clinical diagnosis and evaluation of the effects of tau-targeted treatments.

Fluorine-18 (^18^F) is an attractive radionuclide due to its high positron decay ratio (97%), relatively short half-life (109.7 min), and low positron energy (maximum 0.635 MeV). The positron energy results in a short diffusion range (<2.4 mm), which favorably increases the resolution limits of PET images in clinical and preclinical studies. Based on the pharmacokinetics (5) of scFv in the brain from our previous study, PET imaging with F-18 is, in principle, feasible (11, 15).

Our previous method for labeling bispecific antibody ligands for Aβ was to first produce functionalized antibody ligands with *trans*-cyclooctene (TCO) groups, which in turn were coupled to ^18^F-labeled tetrazines by inverse electron demand Diels–Alder (IEDDA) reactions performed in aqueous solutions (15). Three different ^18^F-labeled tetrazines were synthesized and used in the coupling reactions. *Ex vivo* imaging studies were compared in an Aβ mouse model (tg-ArcSwe) and wild-type control mice. This approach involves several preparation steps, e.g., the antibody must be initially modified, which requires multi-step handling and manipulation of the antibodies prior to the coupling reaction with ^18^F-labeled tetrazines. Therefore, an alternative method was investigated that provides additional possibilities for ^18^F labeling of protein tracers.

The use of the *N*-succinimidyl-4-[^18^F]fluorobenzoate ([^18^F]SFB) prosthetic group to introduce the F-18 radiolabel via various strategies and synthesis modules, followed by purification with either a single or multiple cartridges in series or semi-preparative high-performance liquid chromatography (HPLC), has been reported (16–22). Vaidyanathan and Zalutsky also prepared ^18^F-labeled antibody fragments using [^18^F]SFB, which reacts with the *ε*-amino group of surface-exposed lysine residues on proteins (23, 24). Labeling using this approach showed no loss of affinity for the antibody fragment. Tang et al. further improved the preparation procedure for [^18^F]SFB, which consists of F-18 radiolabeling of the precursor ethyl 4-(trimethylammonium triflate) benzoate, followed by hydrolysis to form a 4-[^18^F]fluorobenzoate salt ([^18^F]FBA) and active esterification to form [^18^F]SFB in a single reaction vessel to further reduce the total synthesis time (25). We, therefore, adapted this procedure using an automated synthesis module (TRACERlab FXFN synthesizer, GE Medical Systems).

**Figure 1:**
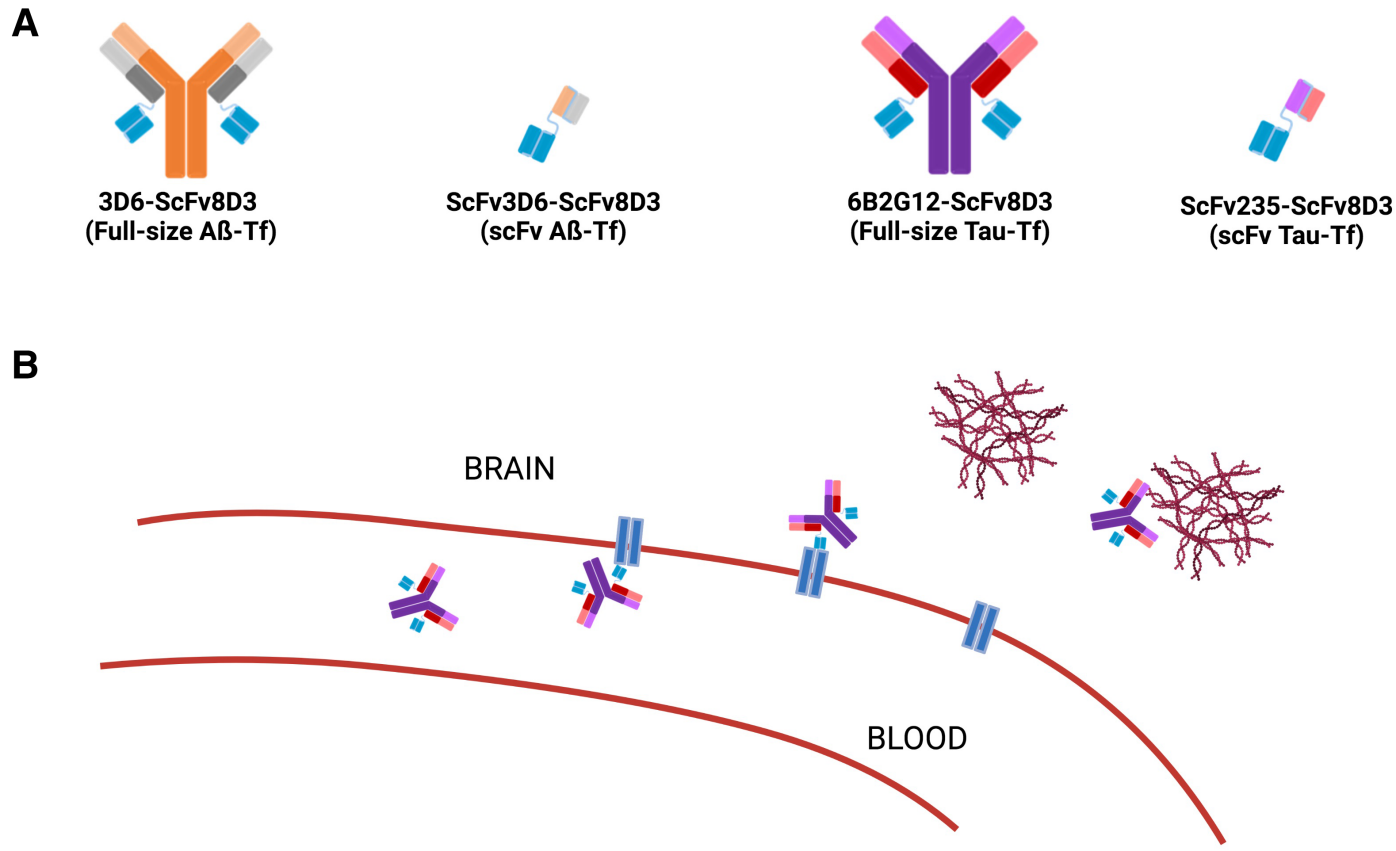
Schematic representation of the bispecific antibody construct. (**A**) The four antibodies produced contain a TfR-specific linker (ScFv8D3, blue) and a variable region specific for either Aβ (full-size: 3D6 or fragment: scFv3D6, orange) or tau (full-size: 6B2G12 or fragment: scFv235, purple). As shown in (**A**), the four bispecific antibody constructs are 3D6-ScFv8D3 (full-size Aß-TfR); ScFv3D6-ScFv8D3 (scFv Aß-TfR); 6B2G12-ScFv8D3 (full-size Tau-TfR); and ScFv235-ScFv8D3 (scFv Tau-TfR). (**B**) Depiction of brain penetration via TfR-mediated transcytosis and specific binding to Aβ or tau protein.

This study aimed to develop methods to perform F-18 radiolabeling with [^18^F]SFB of four specifically engineered novel bispecific antibody ligands, synthesized by fusion of fragments of the TfR with either full-size IgG antibodies of Aβ or tau, or with their respective single-chain variable antibody fragments (scFv of Aβ or tau) (Figure 1), to evaluate their feasibility as PET radioligands for the *in vivo* imaging of Aβ protofibrils and tau protein in the brains of AD mice. Their specificity and ability to detect Aβ or tau aggregates *in vivo,* with distinct quantitative and visual differences in brain uptake between wild-type and transgenic mice, and the correlations between brain uptake and Aβ or tau pathology, can thus be determined and characterized.

## 2. Materials and methods

### 2.1. Materials

To improve brain distribution, Aβ or tau antibodies were fused to a TfR antibody fragment (scFv8D3), to allow receptor-mediated transport across the BBB. Four bispecific antibody constructs, abbreviated as 3D6-ScFv8D3 (full-size Aß-TfR; ∼210 kDa), ScFv3D6-ScFv3D6 (small Aß-TfR; ∼58 kDa), 6B2G12-ScFv8D3 (full-size Tau-TfR; 210 kDa), and ScFv235-ScFv8D3 (small Tau-TfR; 58 kDa), were prepared in the laboratories of Syvänen and Sehlins, Uppsala University, Sweden.

The development of the radiosynthesis protocols, F-18 radiolabeling, purification, formulation of the radiolabeled bispecific antibodies, and subsequent evaluation studies via *in vivo* PET imaging were performed at NYU Radiochemistry and NYU Medical Center (New York City, NY, USA).

All chemicals, including HPLC-grade water, acetonitrile (ACN), ethanol solvent, and ACS reagent-grade and anhydrous ≥99% chemicals, including Kryptofix 2.2.2® (K_222_), *N,N,N′,N′-*tetramethyl-O-(*N*-succinimidyl)uranium tetrafluoroborate (TSTU), potassium carbonate (K_2_CO_3_), and trifluoroacetic acid (Reagent Plus®) were purchased from Sigma-Aldrich (St. Louis, MO, USA).

Ethyl 4-(trimethylammonium triflate) benzoate precursor was synthesized at NYU Langone Medical Center (New York City, NY, USA). The reference standard for [^18^F]SFB (*N*-succinimidyl 4-[^18^F]fluorobenzoate) was purchased from ABX, Germany. Phosphate-buffered solution (PBS) (10×), pH 7.4; Gibco™ was purchased from Thermo Fisher Scientific (Waltham, MA, USA). The sterile water for the injection, USP (for drug diluent use), was purchased from Hospira, Inc. (Lake Forest, IL, USA). Sep-Pak® tC18 Plus short cartridge, Sep-Pak® alumina N-light cartridge, and Sep-Pak® Light, Waters Accell™ Plus QMA cartridge were purchased from Waters (Milford, MA, USA). LiChrolut® SCX (40–63 µm) 200 mg 3 ml standard PP tubes were purchased from Merck & Co. (Rahway, NJ, USA). The Eclipse-HP cyclotron 11 MeV proton beam was provided by from Siemens (Munich, Germany). The F-18 radiolabeling processes were carried out on a GE TRACERlab FXFN auto module (GE Medical Systems, Germany). The quality control analysis was carried out on a Phenomenex RP18 Luna 5 µm 250 × 4.60 mm; the 5-μm column was purchased from Phenomenex, Inc. (Torrance, CA, USA). The quality control HPLC system (Prominence UV/Vis detector, SPD-20A; Communication Bus module, CBM-20A; Prominence Liquid Chromatography LC) was purchased from Shimadzu Scientific Instruments, Inc. (Columbia, MD, USA). The flow-count radio HPLC detector system was purchased from Eckert & Ziegler Radiopharm, Inc. (Hopkinton, MA, USA). The measurement of radioactivity was determined with a CRC 55tR PET dose calibrator (Capintec, Ramsey, NJ, USA). The aluminum thin-layer chromatography (TLC) plate and silica gel coated with fluorescent indicator F254 were from Millipore Sigma (USA). The pH indicator strips were from Sigma-Aldrich (USA). The PD MiniTrap Sephadex G-25 resin size-exclusion column (Cytiva, formerly GE Healthcare) was from Sigma-Aldrich (USA).

### 2.2. Methods

#### 2.2.1. Preliminary *in vitro* and *ex vivo* evaluation experiments

We designed and synthesized novel bispecific tau antibodies according to similar procedures previously published for the Aβ-TfR IgG-based antibody (26) and the smaller tandem single-chain fragment variable (scFv) construct (27). That is, the amino-terminal amino acid sequence of the murine TfR binder scFv8D3 (28) was recombinantly fused via a short linker to the C-terminal end of each of the light chains of the anti-tau IgG antibody 6B2G12 (11) on a mouse IgG2c backbone (29), to generate the full-size Tau-TfR. Further, based on our extensive characterization in live tauopathy mice, we have demonstrated that a lead scFv (scFv235) possessed desired the binding properties to tau protein (11). Thus, the tau binder scFv235 was also recombinantly fused via a standard linker to scFv8D3, resulting in the tau-TfR bispecific tandem (scFv235-scFv8D3) (Figure 1). Using a previously described protocol (30), all these constructs were produced in Expi293 cells and purified using protein G or IMAC (immobilized metal affinity chromatography) columns on an ÄKTA chromatography system. The buffer was exchanged for PBS, and the proteins were concentrated, aliquoted, and stored at −80°C until use.

For both *in vitro* binding and *ex vivo* evaluation experiments, antibodies were radiolabeled with iodine-125 (^125^I) using the chloramine-T method (26, 28). Briefly, 250 pmol of antibody was mixed with ^125^I stock solution (0.108 mCi) and chloramine-T (5 µg) in PBS in a total volume of 110 µl. After 90 s, the reaction was quenched with sodium metabisulfite (10 µg), and the labeled protein was purified from free iodine with a NAP-5 column.

The *in vitro* binding of I-125 radiolabeled and non-labeled antibodies (6B2G12, 6B2G12-scFv8D3, scFv235-scFv8D3) to their respective antigens, TfR, tau peptide 379–408 (abbreviated as tau), and p-tau peptide 379–408 (p-Ser396, 404) (abbreviated as p-tau), was assessed with indirect ELISA before and after radiolabeling. In short, 96-well half-area plates (Corning Inc.) were coated with TfR (1 µg/ml; in-house produced), tau (0.5 µg/ml for IgG, 5 µg/ml for di-scFv235-8D3), or p-tau (0.5 µg/ml for IgG, 5 µg/ml for di-scFv235-8D3) in PBS and incubated at 4°C overnight, then blocked with 1% BSA in PBS. Antibodies, serially diluted from 50 nM, were applied and incubated overnight at 4°C. IgG antibodies were detected with HRP-conjugated anti-mouse IgG F(ab’)_2_ (Jackson ImmunoResearch Laboratories, West Grove, PA, United States) and di-scFv235-8D3 with HRP-conjugated anti-His-Tag antibody (Proteintech Group Inc., IL, USA). Signals were developed with K-Blue aqueous TMB substrate (Neogen Corp., Lexington, KY, USA) and analyzed at 450 nm with a spectrophotometer. All antibody dilutions were made in an ELISA incubation buffer (PBS, 0.1% BSA, 0.05% Tween-20).

For the *ex vivo* evaluation experiments, female C67Bl6 mice (aged 4 months; Taconic Bioscience) were used. Mice (*n* = 4 per group) were injected intravenously with either [^125^I]I-6B2G12 (tau-specific IgG), [^125^I]I-6B2G12-ScFv8D3 (tau-TfR bispecific IgG), or [^125^I]I-scFv235-scFv8D3 (tau-TfR bispecific fragments) via the tail vein at an approximate dose of 6.75 µCi and 37.5 pmol (150 µl of a 250 nM solution). All animals were euthanized at either 2 h or 72 h post-injection through transcardial perfusion with saline. Blood samples were obtained from the heart before the perfusion. The isolated whole brain was divided into the right (RH) and left (LH) hemispheres. The left hemisphere was further divided into the cerebellum (referred to as the cer) and the rest of the brain (referred to as the brain). Radioactivity was measured using a gamma counter (2480 Wizard; PerkinElmer, Waltham, MA, USA).

#### 2.2.2. Radiosynthesis of [^18^F]SFB

Aqueous [^18^F]fluoride was obtained via the nuclear reaction ^18^O(p,n)^18^F by the irradiation of an ^18^O-enriched water target with an Eclipse-HP cyclotron 11 MeV proton beam and was trapped on a Sep-Pak light QMA cartridge. The cartridge was preconditioned with 5 ml of 1.0 M K_2_CO_3_, followed by 6 ml of deionized water (DW). [^18^F]Fluoride ([^18^F]F^−^) was eluted with a 1.5 ml mixed solution (3 mg K_2_CO_3_ in 0.5 ml DW mixed with 15 mg K_222_ in 1.0 ml ACN) transferred to the reaction vessel and evaporated at 110°C for 10 min to produce the anhydrous K_222_/K [^18^F]F complex.

Ethyl 4-(trimethylammonium triflate) benzoate 1 (5.0 mg, 20 µmol) in 1 ml of anhydrous ACN was added to the dried K_222_/K [^18^F]F, and the mixture was heated at 90°C for 10 min to produce ethyl 4-[^18^F]fluorobenzoate 2. The ethyl ester was then hydrolyzed with a 1.0 M tetrapropylammonium hydroxide solution (20 µl) in 1 ml of ACN and heated to 120°C for 3 min under N_2_ and anhydrous conditions. The residue was cooled to 90°C for 3 min to form 3. A solution of TSTU coupling agent (12 mg, 33 µmol) in 1 ml of anhydrous ACN was added and heated to 90 °C for 5 min to form 4. The reaction was cooled to 40°C and immediately quenched with 5% aqueous acetic acid (5 ml) with stirring for 1 min. The crude solution was passed through a tC18 Sep-Pak cartridge (preconditioned with 5 ml of ethanol and 10 ml of DW) and a Sep-Pak alumina cartridge (preconditioned with 10 ml of DW) in series. The reactor was rinsed with DW (12 ml) and again passed through both cartridges. Finally, the cartridges were washed with 10% aqueous ACN (15 ml) and then the product [^18^F]SFB was eluted with 1 ml of ACN. The solvent was evaporated to obtain the dry [^18^F]SFB **4**.

The radiochemical purity of [^18^F]SFB was determined by analytical HPLC on a Phenomenex Luna C18 column (5 µm; 4.6 × 250 mm) at a flow rate of 2 ml/min using the isocratic method (DW: ACN:0.1% TFA; 70:30) and by radio-TLC developed with ethyl acetate as the mobile phase.

#### 2.2.3. General procedure: conjugation of [^18^F]SFB-bispecific antibody reaction

A bispecific antibody (100 µg; 3D6-ScFv8D3, full-size Aß; ScFv3D6-ScFv3D6, small Aß-Tf; 6B2G12-ScFv8D3, full-size Tau-Tf; scFv235-scFv8D3, small Tau-Tf antibodies) was taken into a glass vial containing 0.1 M borate buffer solution (pH 8.5), to which was then added an aliquot of purified [^18^F]SFB in ACN. The mixture was vortexed, checked for a final pH of 8.0, and incubated at ambient temperature for 20 min. The appearance of the reaction mixture was a clear yellow. The conversion and radiochemical yield (RCY) were determined by monitoring the consumption of [^18^F]SFB to form the desired product by radio-TLC using ethyl acetate as the mobile phase. The resulting product was purified by a size-exclusion PD MiniTrap G-25 gravity column preconditioned with a 1× PBS solution. The sample was eluted with 1× PBS; each fraction (0.25 ml) was collected and analyzed by radio-TLC using ethyl acetate as the mobile phase.

## 3. Results and discussions

### 3.1. Preliminary *in vitro* and *ex vivo* evaluation experiments

#### 3.1.1. *In vitro* binding experiments

**Figure 2:**
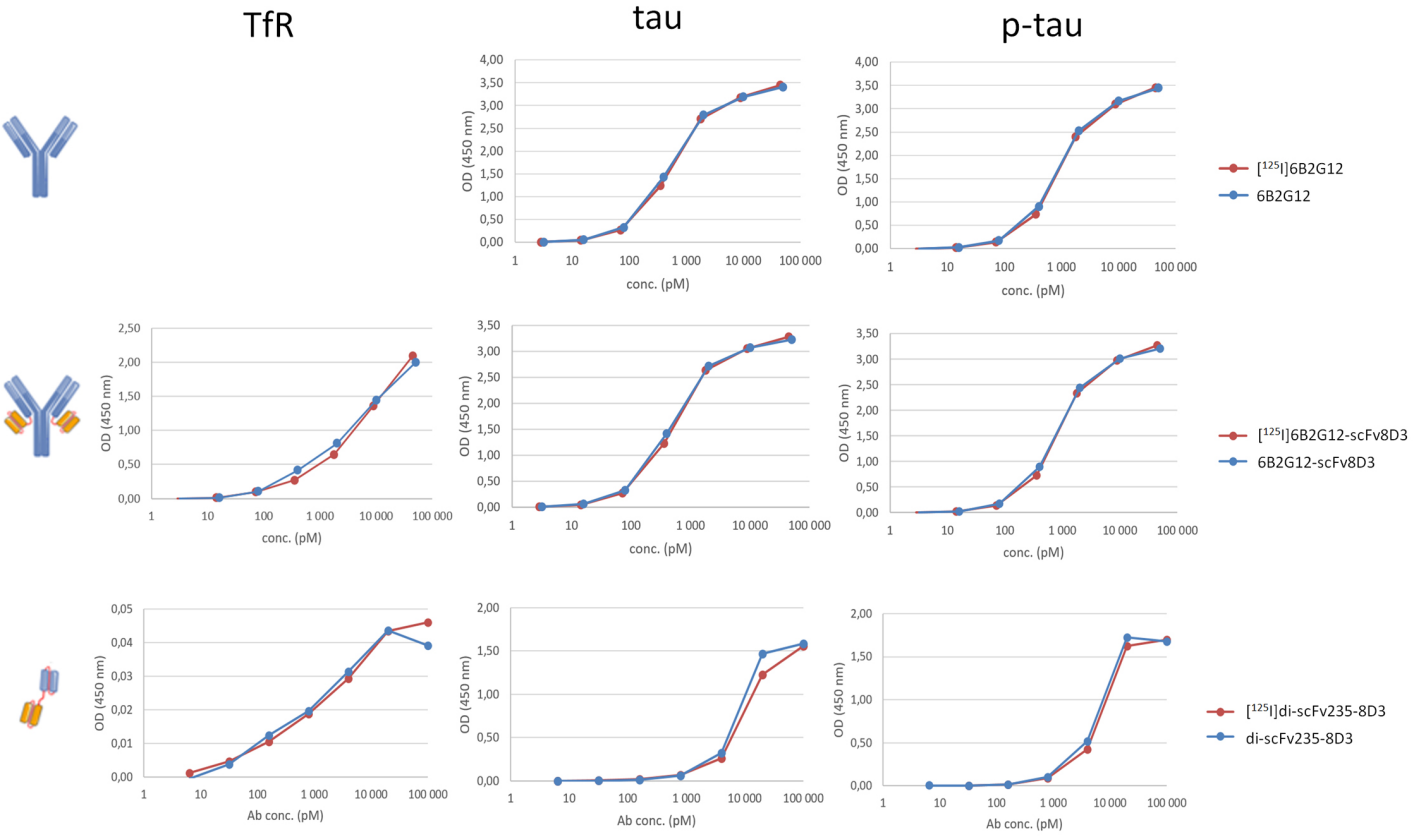
*In vitro* binding of I-125 radiolabeled vs. non-labeled bispecific tau antibodies, such as 6B2G12, 6B2G12-ScFv8D3, scFv235-scFv8D3, was evaluated with TfR, tau, and p-tau ELISA.

For the *in vitro* binding experiments, the binding of I-125 radiolabeled and non-labeled antibodies (6B2G12, 6B2G12-scFv8D3, scFv235-scFv8D3) to their respective antigens, TfR, tau, and p-tau peptides, was investigated via ELISA. The results (Figure 2) are summarized below:

There was no difference in binding properties between I-125 radiolabeled and non-radiolabeled antibodies to their respective antigens, suggesting that the introduction of I-125 did not impact the binding properties. Binding to tau and p-tau peptides was similar for 6B2G12 (IgG) and its bispecific variant 6B2G12-scFv8D3, suggesting that the addition of scFv8D3 (TfR) did not affect binding to its primary target. TfR binding of 6B2G12-scFv8D3 (full-size Tau-TfR) was similar to previous experiments with a similar construct, an Aβ-TfR IgG-based antibody (26).

The tau antibody fragment scFv235 is selective for p-tau over tau under native conditions in solution (11). However, in this experiment, scFv235-scFv8D3 (small Tau-TfR) was applied to an excess of immobilized p-tau and tau peptides on the ELISA plate, which resulted in similar binding to both peptides, as we have shown previously for the parent antibody 6B2G12, which also preferentially binds to p-tau in solution (31). ELISA methods may potentially be optimized to investigate this in future experiments. Low signals in the TfR ELISA of scFv235-scFv8D3 could be due to the suboptimal secondary antibody performance.

#### 3.1.2. *Ex vivo* evaluation experiments

**Figure 3:**
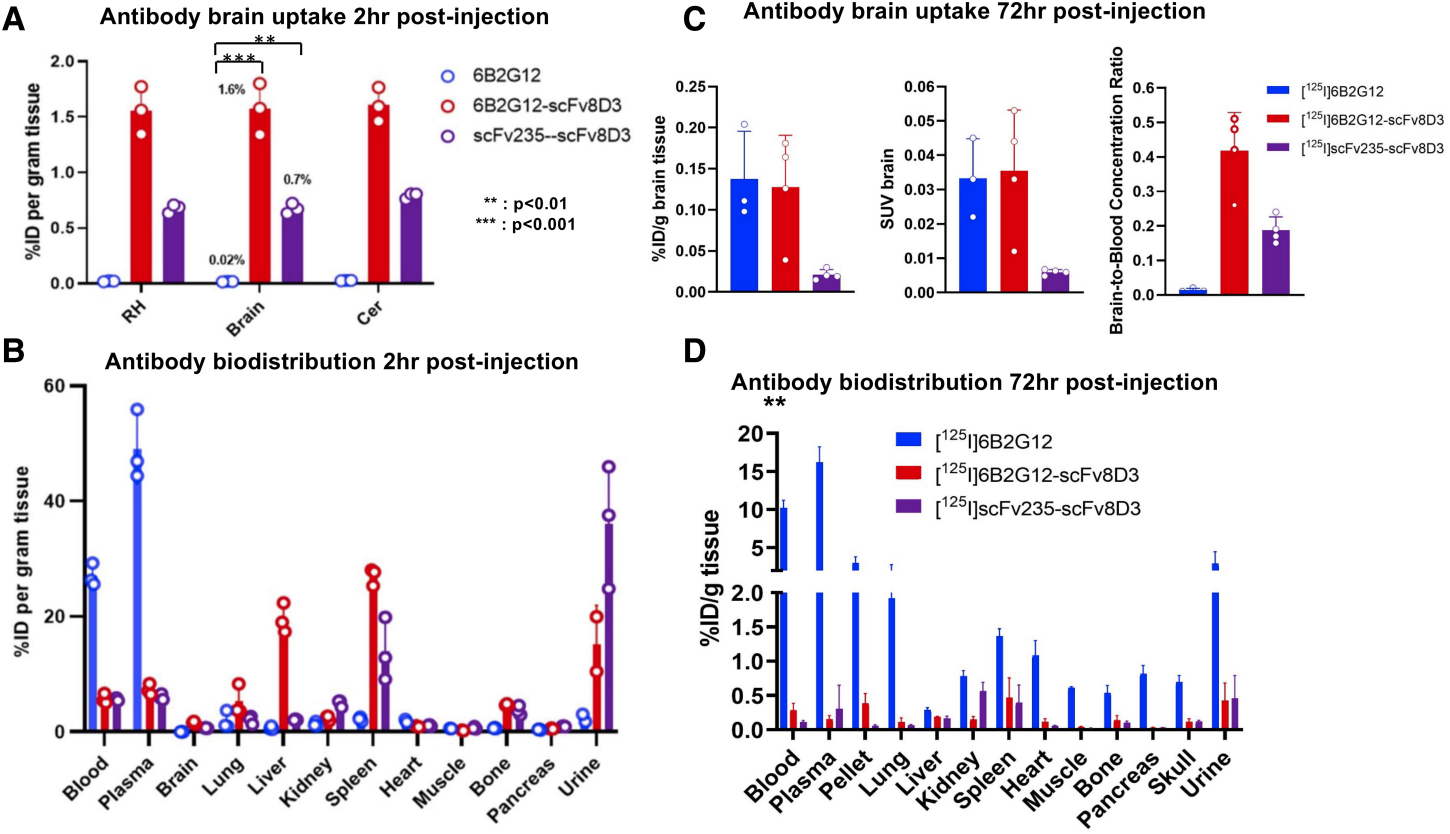
*Ex vivo* study at 2 h (**A**, **B**) and 72 h (**C**, **D**) post-administration of I-125 radiolabeled antibodies 6B2G12 (IgG), 6B2G12-scFv8D3 (full-size Tau-Tf), and scFv235-scFv8D3 (scFv Tau-Tf). (**A**) The two bispecific antibodies displayed much higher brain concentrations than the unmodified antibody (*p* < 0.0001, ANOVA). The brain concentration, quantified as the percentage of injected dose per gram of tissue (% ID/g), was 0.02% for [^125^I]6B2G12 (IgG), 1,6% for [^125^I]6B2G12-scFv8D3 (full-size Tau-Tf], and 0.7% for [^125^I]scFv235-scFv8D3 (small Tau-Tf) at 2 h post-injection (*p* < 0.001 between [^125^I]6B2G12 vs. [^125^I]6B2G12-Scfv8D3; and *p* < 0.01 between [^125^I]6B2G12 vs. [^125^I]scFv235-scfv8D3, Tukey's HSD tests). (**B**) The two bispecific antibodies showed greater accumulation in tissues and excretion organs (liver, spleen, and urine), while the unmodified antibody stayed sequestered in the bloodstream at 2 h post-injection. (**C**, **D**) At 72 h post-injection, the bispecific antibodies had in general lower concentrations in all organs/tissues than the unmodified antibody, except the brain-to-blood ratio remained higher than the unmodified antibody. Tissue concentration was quantified as the percent injected dose per gram of tissue (% ID/g).

For the *ex vivo* evaluation experiments, at 2 h post-injection, the two bispecific antibodies displayed much higher brain concentrations than the unmodified antibody. The brain concentration quantified as the percent of injected dose per gram of tissue (% ID/g) was 0.02% for [^125^I]6B2G12 (IgG), 1.6% for [^125^I]6B2G12-scFv8D3 (full-size Tau-Tf), and 0.7% for [^125^I]scFv235-scFv8D3 (small Tau-Tf) (Figure 3A). In contrast, the bispecific antibodies had considerably lower blood concentrations but higher spleen concentrations compared to the unmodified antibody. Liver concentration was higher for the IgG-like bispecific antibody compared to the other bispecific antibodies, whereas the urine concentration was higher for the tandem-scFv construct compared to the other two constructs (Figure 3B).

A one-way ANOVA was performed to compare the relative brain uptake of the three antibodies. The ANOVA analysis revealed a statistically significant difference in mean uptake between at least two groups (*F*(2, 6) = 1,066, *p* < 0.0001). Tukey's HSD test for multiple comparisons found that the mean uptake was significantly increased in the two bispecific antibodies compared to the unmodified antibody; specifically, a mean difference of 1.557% ID/g (95% CI: 1.453–1.661) for [^125^I]6B2G12 versus [^125^I]6B2G12-Scfv8D3 (*p* < 0.001), and a mean difference of 0.691% ID/g (95% CI: 0.588–0.795) for [^125^I]6B2G12 versus [^125^I]scFv235-scfv8D3 (*p* < 0.01).

At 72 h post-injection, the bispecific antibodies had, in general, lower concentrations in all organs/tissues than the unmodified antibody, suggesting that the two bispecific constructs had a faster clearance from the body than the unmodified full-sized IgG antibody (similar to what we have seen before with other Aβ constructs) (9). However, the brain-to-blood ratio remains higher than that of the unmodified antibody (Figures 3C, D).

The results from both the *in vitro* and *ex vivo* evaluations provided evidence that the bispecific antibodies displayed much higher brain concentrations than the unmodified antibodies and supported the rationale for our development of probes for the *in vivo* PET imaging of tauopathy. Thus, the radiosynthesis of F18-radiolabeled bispecific antibodies using [^18^F]SFB was carried out.

### 3.2. Radiosynthesis and purification of [^18^F]SFB

**SCHEME 1:**
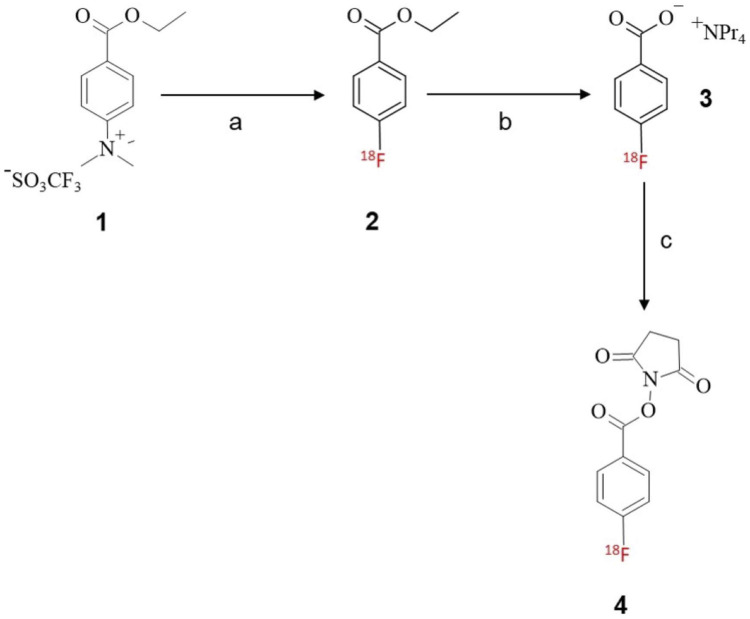
Radiosynthesis of [18F]SFB prosthetic group; reagents and conditions: (**A**) K/K [18F]F, ACN, 90°C, 10 min; (**B**) tetrapropylammonium hydroxide, ACN, 120°C, 3 min; (**C**) TSTU, ACN, 90°C, 5 min.

We adapted the previously reported [^18^F]SFB method with modifications using the TRACERlab FXFN synthesizer (Scheme 1). Briefly, ^18^F-radiolabeling on the ammonium triflate precursor **1** in the solvent ACN afforded **2**, followed by the hydrolysis reaction with aqueous tetrapropylammonium hydroxide to yield the [^18^F]FBA salt **3**. In the final step, the carboxylic acid of [^18^F]FBA **3** is conjugated with the *N*-hydroxy succinimide (NHS) group from TSTU, followed by purification using a Sep-Pak® tC18 plus short cartridge and a Sep-Pak alumina N-light cartridge in series to yield the purified [^18^F]SFB **4**. Detailed investigations on cartridge use, eluting solvent, and volume are described below in Sections 3.2.1 and 3.2.2.

#### 3.2.1. Investigation of cartridge use

We investigated different types of cartridges to improve the purification of [^18^F]SFB without using HPLC purification, as an FXN two-pot module is not always available and it is not always feasible to carry out multi-step reactions with a one-pot module. Initially, we implemented the three-series cartridge method (25), with each cartridge performing a particular role in the purification procedure, i.e., the Sep-Pak® C18 plus cartridge for trapping [^18^F]SFB, the Sep-Pak® alumina N-light cartridge for trapping F-18 free form, and the LiChrolut® SCX (200 mg) cartridge tube for trapping the impurities and byproducts. The purity of [^18^F]SFB was evaluated by an analytical HPLC, which indicated a high radiochemical purity (>99%) but poor chemical purity. Further, using double the amount of SCX packing material (2 × 200 mg) did not improve the purity and resulted in a lower recovery. We also did not find any improvement in reducing impurities with the use of the Sep-Pak® C18 light cartridge.

The Sep-Pak® tC18 Plus short cartridge containing 400 mg of sorbent was then applied for purification. SFB impurities were better removed with a tC18 cartridge compared with an SCX cartridge (400 mg). The results, based on analytical HPLC, suggested that the tC18 cartridge methodology appears to be quite efficient in removing impurities.

#### 3.2.2. Investigation on eluting solvent and volume

The solvent selection and the volume used for eluting [^18^F]SFB from a cartridge are critical to achieving a high percentage recovery of the desired radioactive compound. Rapid evaporation of the solvent from the reaction mixture using a hot plate produced the dry [^18^F]SFB with concurrent cleavage of the succinimidyl group at the carboxylic site to form [^18^F]FBA as a radioactive byproduct.

Several lower boiling points and volatile solvents were investigated, including diethyl ether (20, 32, 33), ethyl acetate (34), chloroform, ACN, and ethanol. Of these solvents, ACN (17, 21, 22, 35, 36) and ethanol (19) both successfully eluted 80%–90% of [^18^F]SFB from the cartridge (Table 1). We also found that restricting the solvent volume to 1–2 ml and reducing the evaporation time to 5 min could produce pure and dry [^18^F]SFB for the subsequent conjugation step with antibodies. Prolonged heating resulted in the decomposition of [^18^F]FBA as a byproduct.

**Table 1:** Comparison of [^18^F]SFB recovery percentages in different solvents. Loading and elution volumes were optimized separately for each solvent for comparison.

S. No	Solvent	Loaded and (eluted) volume (ml)	[^18^F]SFB Recovery %
1	Diethyl ether	3 (0.5)	10–15
2	Ethyl acetate	3 (0.7)	∼50
3	Chloroform	3 (0.5)	10–20
4	Ethanol	2 (1.9)	80–90
5	Acetonitrile	2 (1.8)	80–90

#### 3.2.3. Summarized results on the preparation of [^18^F]SFB

**Figure 4:**
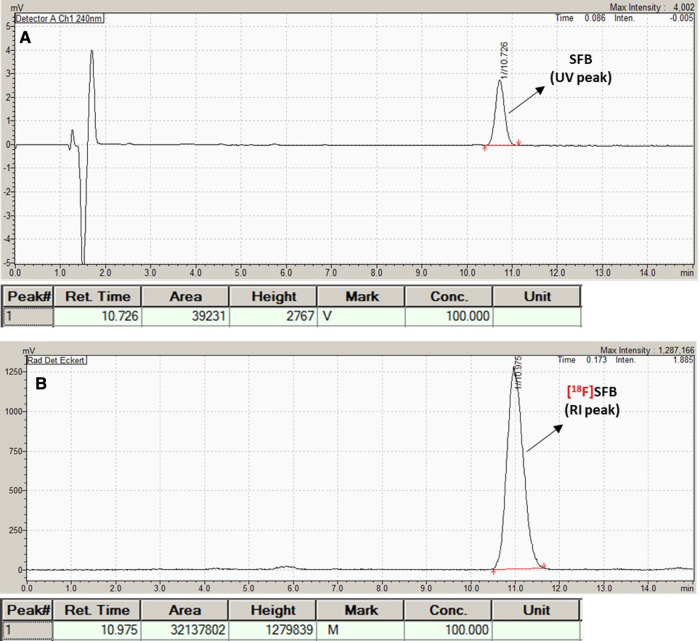
Analytical QC HPLC chromatogram of [^18^F]SFB. (**A**) UV peak (10.726 min); (**B**) radioactive peak (RI).

Radiosynthesis of [^18^F]SFB via a three-step, one-pot procedure accompanying the cartridge purification resulted in a decay-corrected RCY of 46.7% ± 5.4% (*n* = 6) with an overall synthesis time of 65 min, including SPE cartridge purification in the TRACERlab FXN module. The radiochemical purity of [^18^F]SFB was >95% determined both from analytical radio HPLC (Figure 4) and radio-TLC (Rf value of 0.8) (see also Figure 6A). The specific activity (SA) (or molar activity (A_M_)) was 3.15 ± 1.3 Ci/µmol at the end of synthesis (EOS).

### 3.3. Conjugation of [^18^F]SFB with bispecific antibodies to afford [^18^F]SFB-bispecific antibodies

**SCHEME 2:**
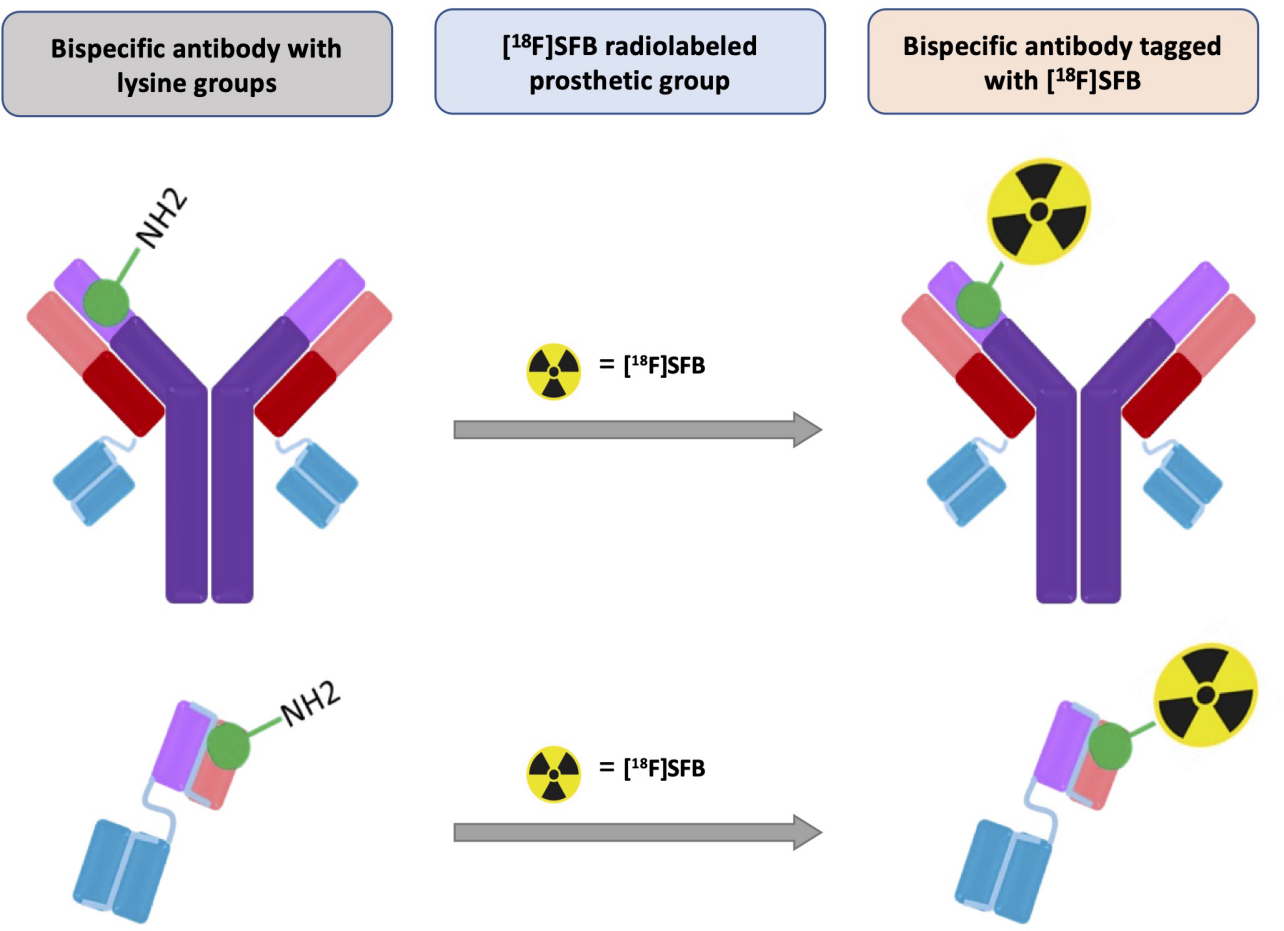
Overview of radiolabeling of bispecific antibodies or fragments via conjugation reaction of [^18^F]SFB to lysine-NH_2_ groups.

An overview of radiolabeling bispecific antibodies or fragments via conjugation reactions of [^18^F]SFB to lysine-NH_2_ groups is shown in Scheme 2.

#### 3.3.1. Investigation of reaction media for [^18^F]SFB-antibody conjugation reactions

To achieve a high radiochemical conversion (RCC), we investigated two conjugation methods to produce the [^18^F]SFB-bispecific antibody. Initially, we performed the conjugation reaction by using [^18^F]SFB with 100 µg of bispecific antibody in a 20% EtOH/1× PBS solution with pH adjusted to 8.0 (2.4 µl 1 N NaOH solution). After incubation at ambient temperature for 20–40 min, radio-TLC showed a low RCC (<35%) to the [^18^F]SFB-bispecific antibody, with 30%–40% of unreacted SFB remaining in the reaction mixture. Poor solubility may be the reason for the low RCC, as the appearance of the reaction mixture was a cloudy pale yellow.

The best results for the conjugation reaction were obtained in a 17% ACN/0.1 M borate buffer solution (pH 8.5) with an incubation of 20 min at ambient temperature. Results showed a higher RCC (70%–83%) for the [^18^F]SFB-bispecific antibody, with less than 10%–15% of unreacted SFB after the reaction, which was confirmed by radio-TLC. Based on these findings, we concluded that it is best to conduct the conjugation reaction in an ACN/0.1 M borate buffer solution conditions compared to EtOH/1× PBS solution. We also noted the clear yellow color of the reaction mixture in ACN/0.1 M borate buffer solution. It is important to avoid using an excess amount of ACN solvent in the conjugation reaction (<20%, e.g., 60 µl of ACN in a total reaction volume of 360 µl) as this could cause precipitation and potentially damage the antibody.

#### 3.3.2. Investigation of pH for [^18^F]SFB-antibody conjugation reactions

To improve the RCC %, we also investigated the effects of pH (Table 2). We established that there was no product formation between pH 5.5 and 6.0 and that product conversion gradually increased at pH 7.0 and 8.0. Our results indicated that pH 8.0 is more efficient for the conjugation reaction in ACN/0.1 M borate buffer solution compared to an EtOH/1× PBS buffer solution.

**Table 2:** Radiochemical conversions (%) of the [^18^F]SFB-bispecific antibody reaction at different pH values, compared between reaction solvents.

S. No	pH	Reaction in EtOH/1× PBS buffer solution; RCC (%)	Reaction in ACN/0.1 M borate buffer solution; RCC (%)
1	5.5–6.0	0	0
2	7.0–7.5	20	20–35
3	8.0	33	70–80
4	8.5	35	<65

RCC, radiochemical conversion.

#### 3.3.3. Investigation of the purification methods for [^18^F]SFB-antibody

**Figure 5:**
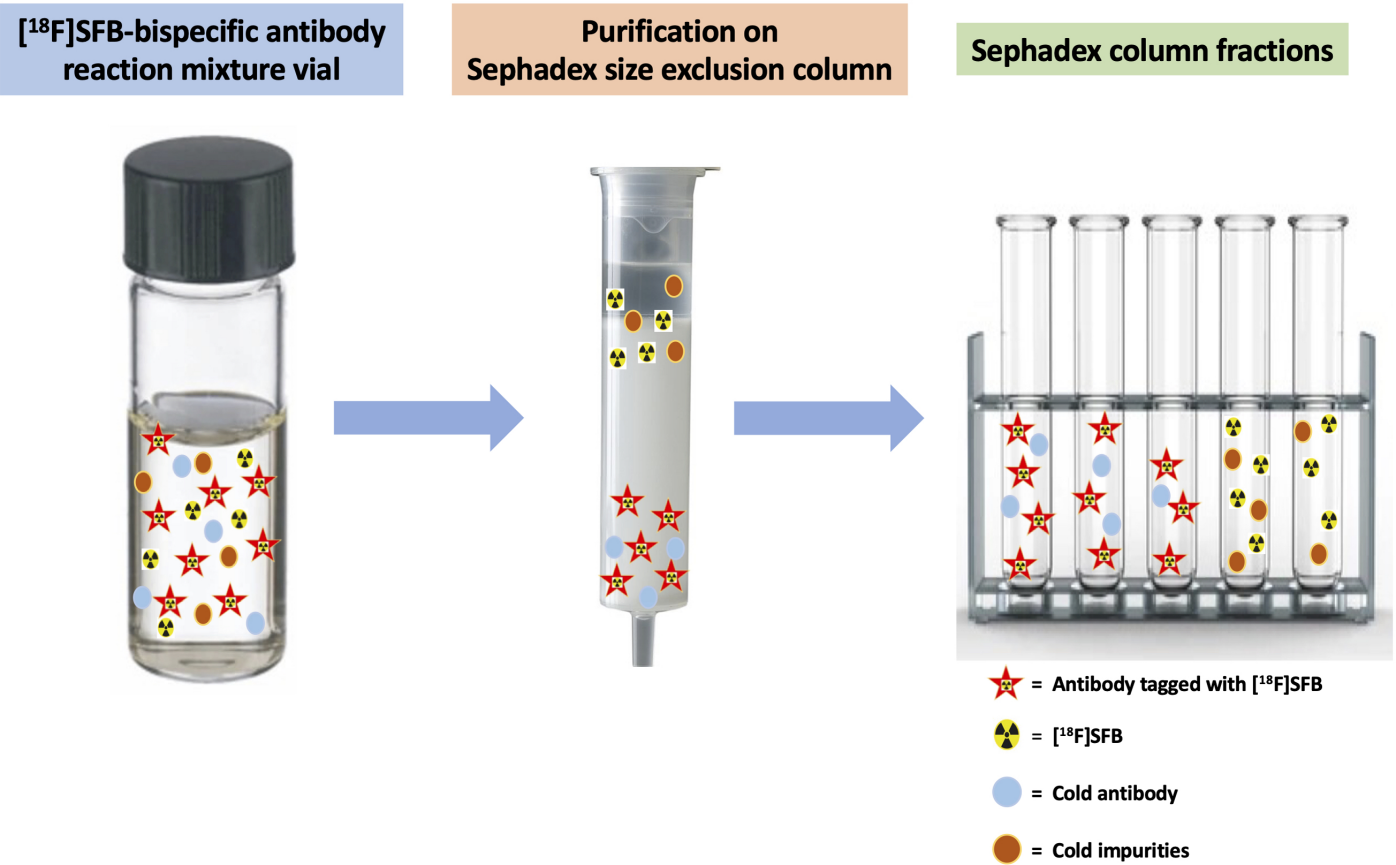
Schematic of [^18^F]SFB-bispecific antibody purification using a PD MiniTrap G-25 size-exclusion gravity column. Separation is achieved on a Sephadex size-exclusion column by elution with 1× PBS. Pure [^18^F]SFB-bispecific antibodies are collected from the earlier fractions, followed by residual [^18^F]SFB in later collections.

**Figure 6:**
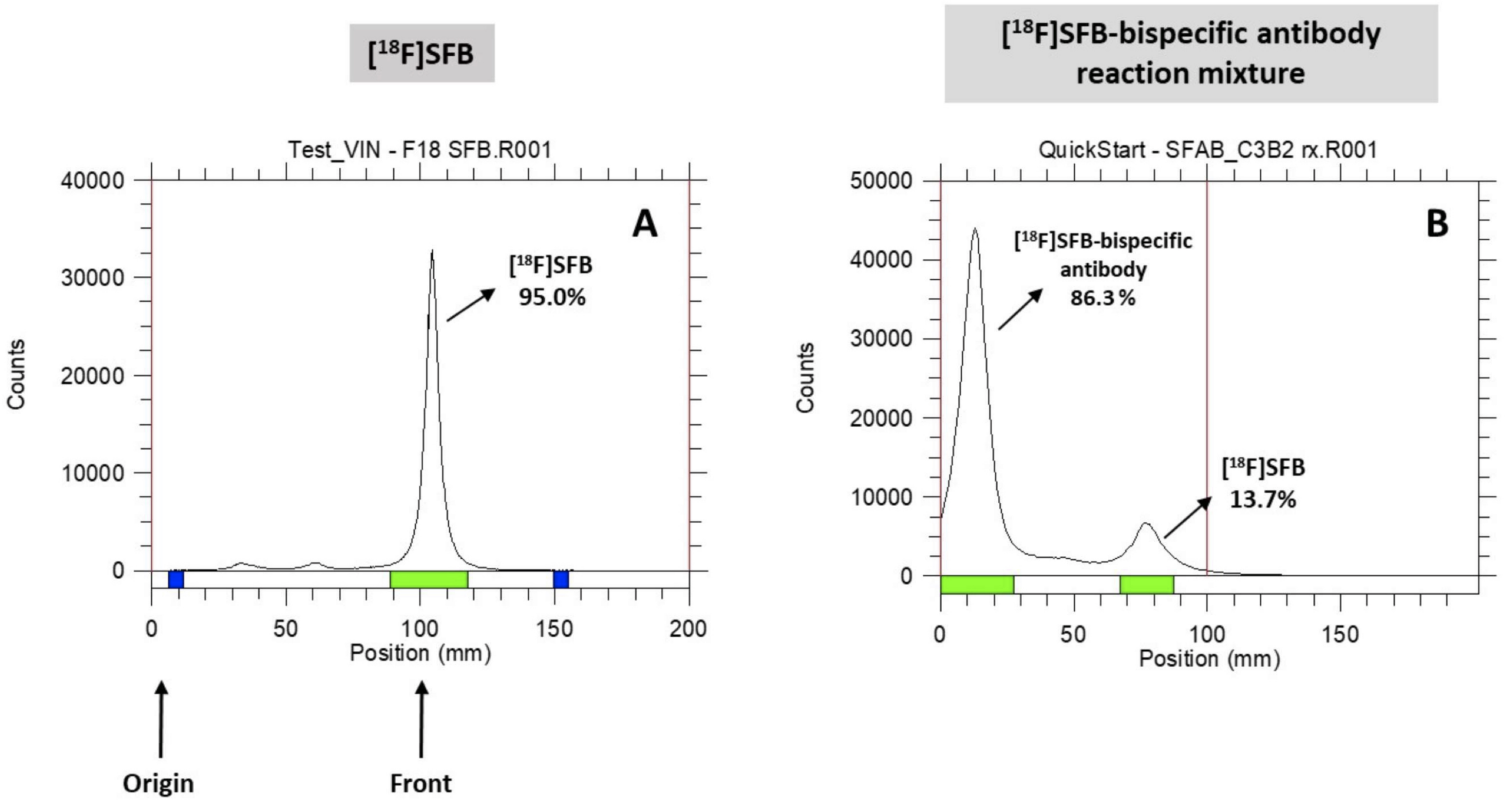
Radio-TLC analysis. (**A**) [^18^F]SFB; (**B**) [^18^F]SFB-bispecific antibody reaction mixture conducted in ACN/0.1 M borate buffer solution.

Various purification methods, such as size-exclusion gravity column chromatography (34, 37, 38), spin columns (39, 40), and spin centrifugal filter units (41), were attempted to purify the [^18^F]SFB-bispecific antibody. Unfortunately, no clear separation of the desired product from unreacted [^18^F]SFB was achieved for all purification methods. The pure [^18^F]SFB-bispecific antibody product was finally isolated by starting with a reduced amount of [^18^F]SFB (≤20× molar excess) for the conjugation reaction and using smaller fraction collecting volumes (0.15–0.2 ml) after loading the reaction mixture onto the size-exclusion gravity column. A diagrammatic presentation of [^18^F]SFB-bispecific antibody purification via a PD MiniTrap G-25 size-exclusion gravity column is shown in Figure 5. Separation was achieved on a Sephadex-size-exclusion column via elution with 1× PBS. The pure [^18^F]SFB-bispecific antibody was collected from the earlier fractions, followed by the remaining [^18^F]SFB in later collections. In the example shown in Figure 6, the reaction mixture was first measured with a CRC 55tR PET dose calibrator and spotted for radio-TLC analysis. With a small fraction collecting volume, the initial fractions eluted from the size-exclusion gravity column contained only the antibody (no SFB) (Rf value for [^18^F]SFB-bispecific antibody = 0.12; free [^18^F]SFB = 0.81); thus, a radiochemical purity of 95%–99% could be achieved. It should be noted that utilizing similar buffer solutions/solvents for the conjugation reaction and the column precondition/elution would render the [^18^F]SFB breakthrough (i.e., it could not be separated from antibodies), thus a different solution/solvent should be used.

#### 3.3.4. Attempted separation of [^18^F]SFB-bispecific antibody from [^18^F]SFB using a glycine quench

In another attempt, we added a glycine solution, which acts as a quencher to abort the conjugation reaction by consuming the excess unreacted [^18^F]SFB. The radio-TLC results indicated the disappearance of the [^18^F]SFB peak ([^18^F]SFB; Rf = 0.81) and the appearance of a new peak at the origin ([^18^F]SFB-glycine; Rf = 0.12), adjacent to the [^18^F]SFB-bispecific antibody (Rf = 0.12). Although glycine can efficiently remove the unreacted [^18^F]SFB, a glycine quench is not recommended as a method of purification due to the poor separation between the radiolabeled glycine ([^18^F]SFB-glycine) and antibody ([^18^F]SFB-antibody) that have similar Rf values.

#### 3.3.5. Summarized results on the conjugation of [^18^F]SFB with bispecific antibodies to afford [^18^F]SFB-bispecific antibodies

We successfully conjugated the [^18^F]SFB with four novel bispecific antibodies (both full-size and scFv versions) in a 17% ACN/0.1 M borate buffer solution (pH 8.5) and incubated for 20 min at ambient temperature. Results showed a higher radiochemical conversion (70%–83%) of [^18^F]SFB-bispecific antibody and 10%–15% of unreacted SFB after the reaction, which was confirmed by radio-TLC (Figure 6). The radiochemical purity of the final product (F)SFB-bispecific antibody) was in the range of 95%–99%. The results of the four probes obtained after being purified by the size-exclusion PD MiniTrap G-25 gravity column (EOS) are listed below (Table 3):

RCY of 51.40% ± 6.10% and SA of 19.60 ± 0.14 Ci/µmol for [^18^F]SFB-3D6-ScFv8D3 (full-size Aß) (*n* = 3);
RCY of 54.15% ± 11.80% and SA of 6.99 ± 1.88 Ci/µmol for [^18^F]SFB-ScFv3D6-ScFv3D6 (small Aß-TfR) (*n* = 4);
RCY of 30.50% ± 4.75% and SA of 24.00 ± 0.17 Ci/µmol for [^18^F]SFB-6B2G12-ScFv8D3 (full-size Tau-TfR) (*n* = 4);
RCY of 33.26% ± 9.22% and SA of 8.57 ± 3.06 Ci/µmol for [^18^F]SFB- scFv235-ScFv8D3 (small Tau-TfR) (*n* = 9).

**Table 3:** Radiochemical yields and specific activity (molar activity) for four bispecific antibody probes.

Antibody	SA (Ci/μmol)	RCY	No. of productions
[^18^F]SFB-3D6-ScFv8D3 (full-size Aß)	19.60 ± 0.14	51.40 ± 6.10	3
[^18^F]SFB-ScFv3D6-ScFv3D6 (small Aß-TfR)	6.99 ± 1.88	54.15 ± 11.80	4
[^18^F]SFB-6B2G12-ScFv8D3 (full-size Tau-TfR)	24.00 ± 0.17	30.50 ± 4.75	4
[^18^F]SFB- scFv235-ScFv8D3 (small Tau-TfR)	8.57 ± 3.06	33.26 ± 9.22	9

SA, specific activity; RCY, radiochemical yield.

## 4. Conclusions

In conclusion, we successfully synthesized the [^18^F]SFB prosthetic group via a cartridge purification method using an automated FXFN module, with an overall RCY of 46.7% ± 5.4% and high radiochemical purity of >95%.

In our study, we found an efficient method to tag four novel bispecific antibodies with [^18^F]SFB under mild conditions, resulting in high radiochemical yields and purities. The reaction showed the best results in the ACN/0.1 M borate buffer solution under mild conditions while preventing the overconsumption of [^18^F]SFB (≤20× molar excess amount) and restricting the fraction collection volume to 0.15–0.2 ml during the purification on a size-exclusion gravity column.

In future experiments, [^18^F]SFB-bispecific antibody ligands will be evaluated for their specificity and ability to detect Aβ or tau aggregates *in vivo*, both quantitatively and visually. Brain uptake in wild-type and transgenic mice and correlations between brain uptake and Aβ, or tau pathology, are currently under investigation.

## Data Availability

The original contributions presented in the study are included in the article, further inquiries can be directed to the corresponding author.
